# Spatially Resolved Expression of Transposable Elements in Disease and Somatic Tissue with SpatialTE

**DOI:** 10.3390/ijms222413623

**Published:** 2021-12-20

**Authors:** Braulio Valdebenito-Maturana, Cristina Guatimosim, Mónica Alejandra Carrasco, Juan Carlos Tapia

**Affiliations:** 1 Núcleo Científico Multidisciplinario, School of Medicine, Universidad de Talca, Campus Talca, Talca 3460000, Chile; braulio.valdebenito@utalca.cl; 2 Departamento de Morfologia, ICB, Universidade Federal de Minas Gerais, Belo Horizonte 31270-901, Brazil; cguati@icb.ufmg.br; 3 School of Medicine, Universidad de Talca, Campus Talca, Talca 3460000, Chile

**Keywords:** spatial transcriptomics, transposable elements, gene regulation, spinal cord & brain, kidney, amyotrophic lateral sclerosis disease

## Abstract

Spatial transcriptomics (ST) is transforming the way we can study gene expression and its regulation through position-specific resolution within tissues. However, as in bulk RNA-Seq, transposable elements (TEs) are not being studied due to their highly repetitive nature. In recent years, TEs have been recognized as important regulators of gene expression, and thus, TE expression analysis in a spatially resolved manner could further help to understand their role in gene regulation within tissues. We present SpatialTE, a tool to analyze TE expression from ST datasets and show its application in somatic and diseased tissues. The results indicate that TEs have spatially regulated expression patterns and that their expression profiles are spatially altered in ALS disease, indicating that TEs might perform differential regulatory functions within tissue organs. We have made SpatialTE publicly available as open-source software under an MIT license.

## 1. Introduction

RNA-sequencing (RNA-Seq) has transformed the way we can study gene expression and its regulation. This is due to the ability to detect transcripts at a whole-genome scale in an unbiased manner (i.e., it does not require a priori knowledge of which genes to study) [1]. Although the method has expanded the knowledge on mechanisms of gene regulation, in recent years, efforts have been made to obtain transcriptomes with greater cellular resolution, as the standard bulk RNA-Seq protocol represents a transcriptome “average” (i.e., the expression of all cells from the sampled tissue is mixed) [2]. Recently, single-cell RNA-Seq (scRNA-Seq) methods have been developed, allowing a significant advance in terms of the transcripts expressed by specific cell types, further improving gene expression analysis in highly heterogeneous tissues (i.e., brain, spinal cord, kidneys, etc.) [3]. An innovation, parallel to scRNA-Seq is that of spatial transcriptomics (ST), in which transcriptomes are obtained from a well-defined spatial location within the tissue [2]. Briefly, in ST, a tissue section is placed on a glass slide with a grid of spots, each of which has a microarray of oligonucleotides (also known as “capture probes”) that allow the direct capture of the mRNAs from the grids maintaining their spatial position. Then, cDNA libraries are prepared and sequenced using standard DNA sequencing technologies, which in turn allows the estimation of transcriptomic profiles at each spatial spot [1,4]. Currently, there are two generations of ST: the original ST method, developed in 2016, that allows the capture of transcriptomes from spots of 100 μm in diameter, and 200 μm of center-to-center distance between the spots, limited to a total of 1007 spots per slide [2,4]; and the 10X ST that decreases of the spot sizes and the center-to-center distance between them to 55 μm diameter and 100 μm, respectively, increasing the total spots per slide to 5000 [4]. So far, the ST methods have been successfully applied in diseased and healthy tissues. Particularly, the original ST has been used to study the tissue heterogeneity of several cancer types [5,6], along with the fatal neurodegenerative disease amyotrophic lateral sclerosis (ALS) [7]. Thus, it is now possible to assess the spatial activity of genes, a key step in understanding differential gene regulation in highly heterogeneous tissues.

Transposable elements (TEs) are genetic segments that can move and replicate themselves within a genome [8]. TEs are classified into DNA transposons and retrotransposons, the latter further subclassified into the LINEs (long interspersed nuclear elements), SINEs (short interspersed nuclear elements) and LTRs (long terminal repeats). TEs are found ubiquitously in exons, introns and intergenic non-coding regions in almost all genomes; they occupy nearly 50% of the human and mouse genomes. Due to their potentially harmful activity, most TEs are fragmented and carry mutations that render them inactive (labelled as “old TEs”), with only a few amounts of TEs being intact, and highly identical to each other (labelled as “young TEs”) [8]. Because of this, they were originally thought to be junk DNA. However, transcriptional activation of TEs can still occur, and it has been associated with a role in regulating gene expression [8,9]. Several algorithms have been developed to study their expression in bulk RNA-Seq datasets [8]. According to the quantification strategy used, the approaches can be divided into two categories: TEs quantification at the subfamily/family level and at the locus-specific level. The first approaches circumvent the multimapping problem by combining TE counts at the subfamily/family level, resulting in a more accurate estimation of TE expression (in particular for younger TEs), but at the expense of losing their genomic location [8,10]. On the other hand, locus-specific approaches work relatively well for old TEs because they have accumulated enough mutations that differentiate them [11], but have problems with accurately estimating the expression of young TEs, which are often highly similar between them earlier. Locus-specific approaches, however, allow the study of the genomic vicinity of TEs, further helping to understand their influence in genetic programs [12].

Previously, we described the transcriptional landscape of TEs during the progression of ALS using the SOD1^G93A^ mouse as a model [13]. Since the work used bulk RNA-Seq datasets, we were unable to examine the expression of TEs in specific regions of the ALS-afflicted spinal cords. Recently, the Phatnani group performed ST of ALS spinal cords from the same mouse model. Although they described the spatial patterns of expression of genes during the disease progression, the group did not examine TEs expression. In this work, we take advantage of those datasets and others to present SpatialTE, a pipeline to analyze TEs from datasets obtained by either the original ST or the 10X ST approaches. We applied SpatialTE to data from ALS diseased mouse spinal cord and show that TEs are indeed expressed in distinct spatial locations. We expanded the use of SpatialTE to other highly heterogeneous tissues (brain and kidney), in which the 10X ST approach has been used. In these datasets we also found expressed TEs, and that some of them are differentially expressed in specific regions. In sum, our results suggest that TEs might play regulatory roles that depend on the cells and their location within the tissues.

## 2. Results

### 2.1. Spatially Resolved TE Expression of p120 Spinal Cord Sections from SOD1^G93A^ Mice

The SOD1^G93A^ mice is a well-characterized model to study ALS since it accurately recapitulates the disease progression seen in humans (motor neurons death, muscle weakness, and lower limb paralysis at p120) [13,14,15]. The Phatnani group applied the original ST in spinal cords of SOD1^G93A^ mice. Previously, using bulk RNAseq from whole spinal cords, we found differential activation of TE in the SOD1^G93A^ model [13]). However, we were unable to know whether the TE activities were specific to some regions of the spinal cord i.e., to cells present within the spinal cord. With the development of SpatialTE, we expected to overcome these problems and answer three basic questions: (1) Is it possible to determine changes in TEs expression using data generated with the original ST (spinal cord)? (2) Do regions of the ALS spinal cord sections, known to be differentially afflicted by the disease, show differences in the activity of TEs? (3) If so, what subclasses of TEs are spatially transcribed TEs altered in ALS spinal cords?

**Figure 1:**
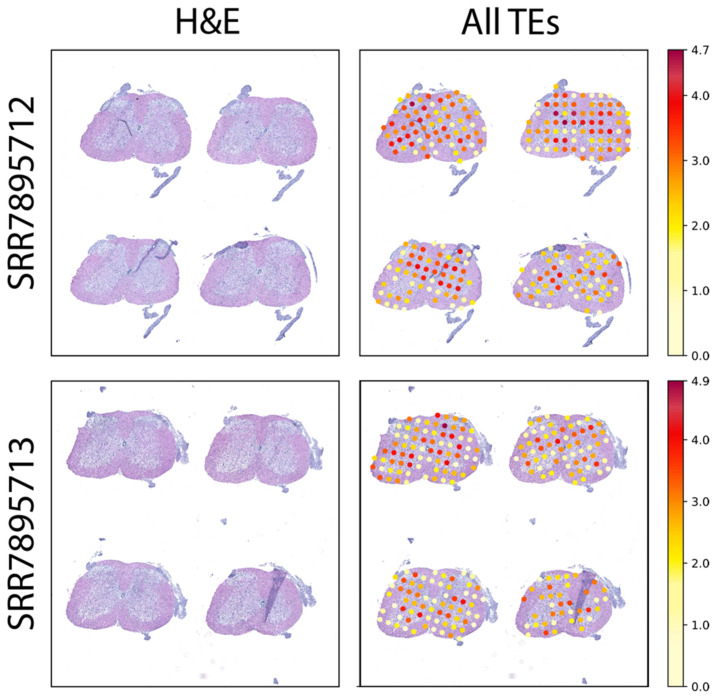
TE expression in the p120 SOD1^G93A^ mice using samples obtained with the original ST. SRA accession corresponding to each sample is indicated at the left; “H&E”: hematoxylin and eosin-stained tissue; “All TEs”: TE expression per spot over the tissue. Expression levels correspond to log2 normalized counts, with the colour scale indicated at the right (lower values in light yellow, with higher values in dark red).

Applying SpatialTE to the original ST datasets from the SOD1^G93A^ spinal cords, we find that TEs are ubiquitously expressed (Figure 1). Interestingly, the expression of TEs was higher in the dorsal and ventral horns, than the expression observed in more medial or distal regions of the spinal cord (Figure 1, right panels). This is an interesting finding because the cell population most affected during ALS is located within the ventral horn [16].

**Figure 2:**
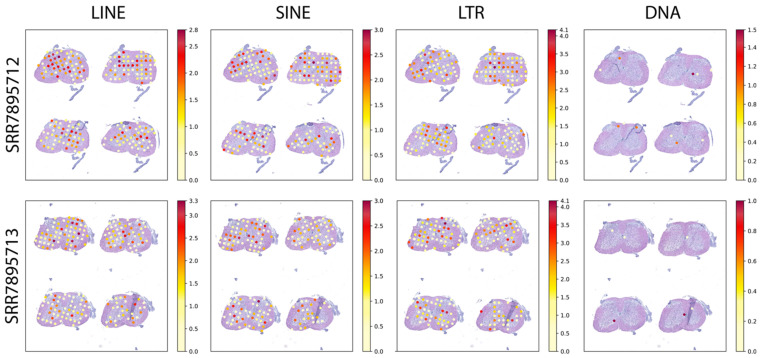
TE expression by class in the p120 SOD1G93A spinal cord. Samples are indicated by their SRA accession to the left, and each TE class is indicated above. Expression levels correspond to log2 normalized counts, with the colour scale indicated at the right of each panel

Then, we analyzed the TE expression according to the categories of LINE, SINE, LTR, and DNA transposons (Figure 2). This analysis revealed that all categories, besides the DNA TEs, contribute to the total TE expression. These results are in agreement with evidence indicating that some LTRs and non-LTR TEs (i.e., LINEs and SINEs) are activated in the disease [17]. Thus, our results reveal differences between the TE classes (Figure 2). The specific influence of different TE classes on ALS disease progression has not been fully elucidated. Here, we show that there are subtle differences between the transcriptional activation of LINE, SINE, and LTR TEs across the spinal cord, allowing us to speculate that particular TEs might influence the disease in different manners. Previous studies have indicated that both LTR and LINE TEs activate during the disease, yet their actual contribution to neurodegeneration has not been fully elucidated [13,18,19]. With our work, we confirm these previous findings and further expand the TE repertoire by showing that SINEs also become transcriptionally activated. Moreover, taking advantage of the spatial resolution, the finding of TEs expressed in the ventral horn regions could further suggest that they might influence neurodegeneration.

### 2.2. Spatially Resolved TE Expression in the Adult Mouse Brain

In addition to our interest in ALS spinal cords, we wanted to examine whether SpatialTE could be used in a highly heterogeneous tissue, such as the brain. We know activation of TEs occurs in the germline during early development [20,21] and also in neurons [22,23,24]. Despite these data, very little is known about the spatial distribution (i.e., expression) of TEs in different regions of the brain. Thus, using SpatialTE on 10X ST datasets from adult mouse brain, we aimed to uncover the TE repertoire and assess their spatial expression across the brain.

**Figure 3:**
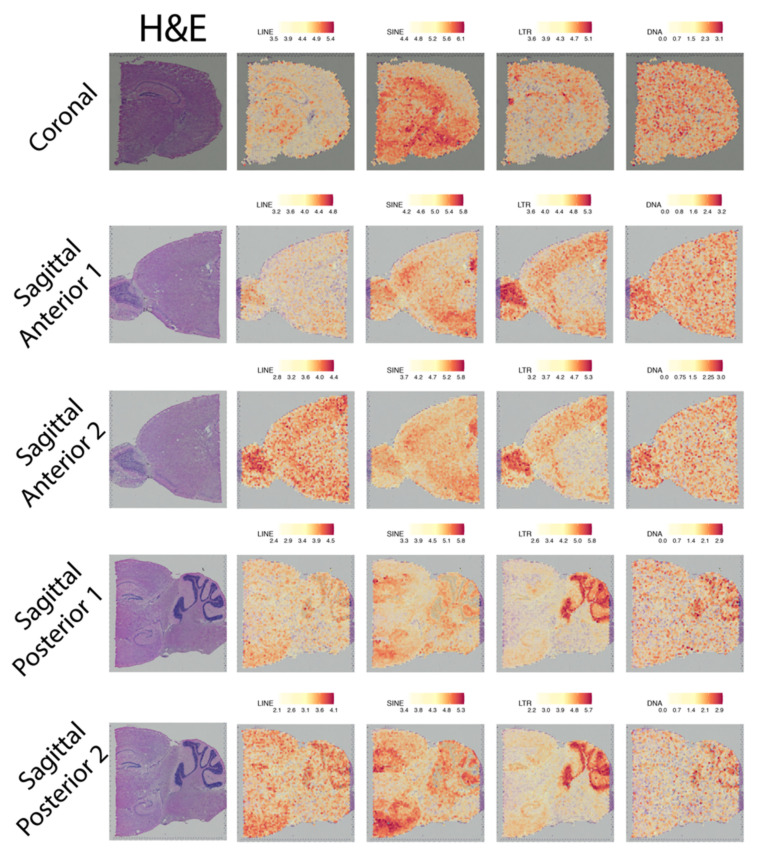
TE expression by class in the adult mice brain. Brain sections are indicated at the left, and the expression of each of the TE classes (indicated above) are shown. Expression levels correspond to log2 normalized counts, with the colour scale indicated at the top of each plot. “H&E”: hematoxylin and eosin-stained tissue.

TE expression could be seen across all brain sections, with each class showing differential patterns of activity (Figure 3, Table 1). In the coronal brain section, there is a marked expression of SINEs at the subcortical areas (Figure 3, top third column), with LINEs showing subtle expression in these regions, but particularly increased levels at the thalamus (Figure 3, top second column). By contrast, LTRs show slightly marked expression in the brain cortex (Figure 3, top fourth column). For sagittal anterior datasets, both SINEs and LTRs show consistent inter-sample expression (i.e., they show similar levels and spatial distribution of expression in both replicas). SINEs are increased in the cerebral cortex, hippocampus and hypothalamus, whereas LTR levels are elevated in the olfactory bulb and cerebral cortex. LINEs on the other hand, show similar patterns of spatial expression but in different magnitudes (less expression in sample 1 than sample 2). Similar to LTRs, the spatial expression of LINEs seems to occur mainly in the cerebral cortex and olfactory bulb. In the sagittal posterior samples, SINEs and LTRs are also the predominant TE classes. SINEs are spatially restricted to the hippocampus, hypothalamus (in agreement with the results from the sagittal anterior samples), and cerebellum, whereas LTRs are strongly expressed in the cerebellum. LINEs expression, albeit not as marked, seems to preferentially occur in the cerebral cortex, hypothalamus, and cerebellum. Surprisingly, we also found DNA TEs expressed. TEs from this class are rarely studied, because they only represent a small fraction of the murine genome (between 1–2%). However, across all brain sections, we found that the DNA TEs were heterogeneously expressed, without an apparent spatial preference, indicating that they are less likely to play a significant role.

In summary, these results indicate that TEs have differential spatial expression, further suggesting that TEs contribute in specific ways to gene regulatory network characteristics of each brain region.

### 2.3. Spatially Resolved TE Expression in the Adult Mouse Kidney

**Figure 4:**
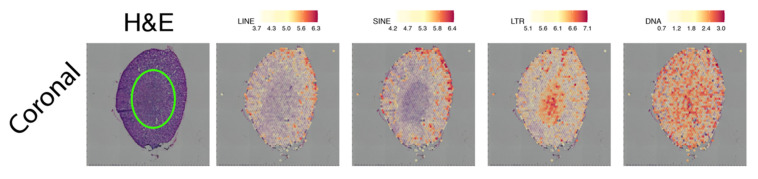
TE expression by class in the adult mice kidney, coronal section. Each TE class is indicated above. Expression levels correspond to log2 normalized counts, with the colour scale indicated at the top of each plot. “H&E”: Hematoxylin and Eosin-stained tissue. A green oval has been added to the H&E figure to highlight the medulla.

Finally, we wanted to use SpatialTE on samples in which TEs have not been studied. For this, we used corresponding coronal sections of the adult mice kidney (Figure 4). To the best of our knowledge, TE expression in healthy kidneys has not been analyzed.

Our results show that DNA TEs seem to have no spatial preferences. Their expression levels, although relatively high, are distributed throughout the kidney (Figure 4). LINEs and SINEs, on the other side show low levels of expression, with a few limited spots in a portion of the cortex (Figure 4, column 3). LTRs are the ones showing the most marked spatial preferences in expression, being predominantly activated in the medulla (Figure 4). This region is among the most important for proper kidney functioning, as its activities involve the modulation of urine concentration [25]. Thus, our results show that indeed TEs are differentially expressed in kidneys, and that their expression, at least for some of the TE, is spatially controlled. Moreover, the results suggest that TEs play a regulatory role in regions of the kidney (medulla vs. cortex).

## 3. Methods

### 3.1. SpatialTE Implementation

**Figure 5:**
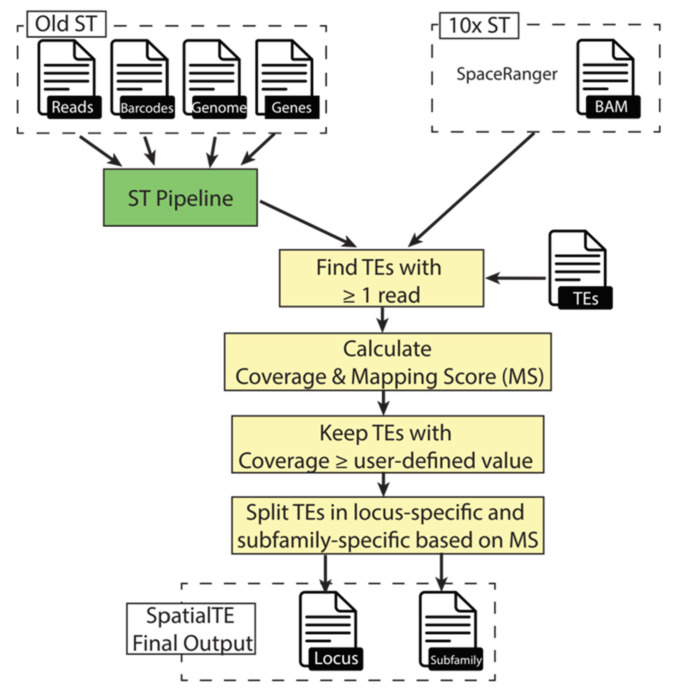
Workflow of SpatialTE. Dashed boxes correspond to the input files (shown with a file icon and a name) for SpatialTE according to the ST technology used (Old or 10×). The green box corresponds to the run of the external tool ST pipeline (which performs read alignment and identification of gene expression per spatial spot), whereas yellow boxes correspond to key processes within SpatialTE. First, TEs having at least 1 read are selected. Second, for these selected TEs two metrics are calculated: Coverage (percentage of TE bases covered by reads) and the mapping score (number of uniquely mapped reads aligned to the TE divided by the total number of reads aligned by the TE). Third, TEs can be filtered by a user-defined coverage threshold (default = 0, i.e., keep all TEs). Finally, SpatialTE generates two outputs according to the mapping score of the TEs (output files, TEs by locus and TEs by classes).

SpatialTE employs a strategic application of several alignment metrics to subsequently assess the expression of TEs from datasets generated using either the original ST or the 10X ST (Figure 5). However, each approach requires different inputs due to the way the files are handled in their corresponding computational tools. To process datasets generated with the original ST, the ST pipeline [26] is employed, generating a matrix that contains the expression of genes from all spatial spots, and a BAM file with the reads that correspond to the exons of annotated genes. On the other hand, the SpaceRanger tool is used for the analysis of 10X ST datasets. It generates a gene expression matrix and a BAM file that contains all aligned reads. Thus, for SpatialTE in the original ST modality, a customized call of the ST pipeline is done to keep all aligned reads, and for the 10X ST modality, it starts directly from the BAM file. Regardless of the modality, a TE annotation file is required, such as the one obtained with RepeatMasker (the gold standard tool to annotate transposable and other repetitive elements). The RepeatMasker annotation files for several organisms (such as *Homo sapiens*, *Mus musculus*, and others) are readily available at the UCSC genome browser database [27] and the NCBI Genome database. SpatialTE is packed with a script that conveniently transforms the output of RepeatMasker to the appropriate format. The bases for SpatialTE are all the reads that are mapped to non-exonic regions of the genome, which are not further considered in the standard ST analysis.

Due to their repetitive nature, the main problem for examining TE expression with any type of RNA-Seq data are multi-mapper reads. Although, old TEs have accumulated mutations [11] that make possible a unique alignment of reads to them. The second step in SpatialTE is to find which TEs have at least 1 read. Then, for these TEs, two metrics are calculated: coverage and mapping score.

The coverage is defined as the percentage of the TE bases covered by reads and is calculated using BEDTools [28]. All TEs with a coverage equal to or above a user-defined threshold are kept (default value = 0, i.e., all results are reported). Higher coverage values can be used to detect full-length TEs, while lower coverage values can be used to include shorter TE transcriptional variants (i.e., TEs having only a fraction of their locus transcribed) [8]. An issue with the coverage metric is that it does not take into account the proportion of unique- and multi-mapper reads. Thus, the mapping score (MS) is calculated afterward. MS for a particular TE_i_ is calculated as the number of uniquely mapped reads (UMRs) to TE_i_ divided by the number of uniquely mapped reads to TE_i_ plus the number of multi-mapped reads (MMRs) to TE_i_:

$$\mathrm{MS}({\mathrm{TE}}_{i})=\frac{{\;\mathrm{UMRs}\;\mathrm{TE}}_{i}}{{\mathrm{UMRs}\;\mathrm{TE}}_{i}+{\mathrm{MMRs}\;\mathrm{TE}}_{i}}$$

In this way, the MS represents a filter for TEs based on the ambiguity of the reads aligning to it. MSs near or equal to 100 would represent TEs whose expression can be more accurately assessed at the locus level, whereas lower MSs represent TEs in which there is less certainty on their expressed levels at a specific locus. Afterward, TEs are split into two groups: locus-specific and subfamily-specific. Locus-specific TEs are those TEs that have an MS equal to or above a user-defined value (default = 100, i.e., TEs having only UMRs), whereas subfamily-specific TEs are TEs having an MS equal or lower than a user-defined value (default = 0, i.e., TEs having only MMRs). Subfamily-specific TEs are further processed to report expression levels summarized at the subfamily level, instead of a locus-specific level, as there is no certainty from which locus a TE with MS = 0 comes from. Finally, for each TE group, the results are provided as a TSV matrix containing the TE identifiers as columns, and spot coordinates as rows. Thus, the results are readily integrable into subsequent analysis by using any of the tools that are publicly available for ST analysis.

SpatialTE is implemented as an open-source Bash script, with instructions detailing its usage available at the README file in the GitHub repository, publicly available at: https://github.com/bvaldebenitom/SpatialTE (accessed on 15 October 2021).

### 3.2. Benchmarking and Validation

Due to the lack of proper ST simulation tools, it is not feasible to extensively test SpatialTE with synthetic data. There are no other tools to study TEs in ST data; thus, we cannot compare our tool to others that could be similar. Nonetheless, we designed a small proof-of-concept experiment using data from a real ST sequencing experiment. First, we took advantage of the fact that ST sequencing is done using a paired-end layout in which spatial information is in read 1 and expressed RNA information is in read 2. We selected all the reads that belonged to 2 spots of the sample SRR7895713 sample: the 11 × 5 and the 10 × 10 spots (Figure S1). Then, we randomly selected LINEs and LTRs matching the available number of molecules at each spot. For each randomly selected TE in the respective spot, a random number of reads was generated using the Polyester RNA-Seq read simulator in single-end mode [29]. For the spot 11 × 5, a total of 6420 reads were generated, whereas for the 10 × 10 spot, a total of 9593 reads were generated (Supplementary Tables S1 and S2). The simulated file was used as the right read, and the spatial information for the respective spots (11 × 5 for LINEs, 10 × 10 for LTRs, Figure S2) was used as the left read. These files were then used as input for SpatialTE. Since the spatial location and molecule information belonged to a real experiment (SRR7895713), we used the respective H&E image to visually inspect the results of SpatialTE. Moreover, because we knew the ground truth spatial position and expression levels of these TEs, we were able to analyze and compare the expression estimates obtained with SpatialTE against the real expression levels. Overall, this experiment allowed the researchers to test the accuracy of SpatialTE in correctly assigning TE expression to each spatial spot, and the accuracy of its expression estimates. With this test, we confirmed that in terms of spatial coordinates, no errors were detected. This indicates that our tool can precisely reveal the location of TE expression across the studied tissues Additionally, we found that there is higher correlation to real expression levels between the expression estimation at the subfamily level than those at the locus-specific level (Figure S3). These tests and their results are further detailed in Supplementary File S1.

### 3.3. SpatialTE Data Analysis

SpatialTE was applied to publicly available datasets generated using either the original ST or the 10X ST. Since all the datasets were obtained from mice, the *Mus musculus* mm10 genome was used along both the gene and transposable element annotation, all obtained from the UCSC genome browser database [27].

The original ST dataset used corresponded to samples generated from the spinal cord of p120 SOD1^G93A^ mice. The raw reads FASTQ files are available at the Sequence Read Archive (SRA accessions SRR7895712 and SRR7895713), while the corresponding hematoxylin and eosin stained tissue images are available at the Gene Expression Omnibus database (GEO accessions GSM3399315 and GSM3399316). The 1000L7 barcode file required to perform spatial demultiplexing of these samples is available at GEO accession GSE120374. SpatialTE was then used in the original ST mode, and the TE Subfamily count matrix by spatial position output was used in the subsequent analysis. Generation of figures depicting TE expression over the H&E images was done using the st_data_analysis tool from the ST Analysis package [26], with options “--image-files” indicating the corresponding H&E jpg file, “--counts” using the SpatialTE output, “--use-log-scale” to report results in log-normalized scale and “--show-genes” indicating the main TE classes LINE, SINE, LTR and DNA.

The 10X ST datasets were obtained from the 10X Genomics Spatial Gene Expression datasets webpage. The datasets used in this work correspond to mouse brain (1 coronal, 2 sagittal anterior, 2 sagittal posterior) and kidney (1 coronal) sections. URLs to each dataset are indicated in Supplementary File S2. SpatialTE was used in the 10X ST mode, and the TE subfamily count matrix by spatial position output was used in the subsequent analysis. TE spatial analysis was done using the R [30] package Seurat v4 [31], using the functions “Load10X_Spatial” to load the data, “SCTransform” to perform count normalization, “RunPCA”, “FindNeighbors”, “FindClusters” and “RunUMAP” collectively to perform dimension reduction, “SpatialDimPlot” to visualize the clusters over the H&E image, and “SpatialFeaturePlot” to visualize the main TE classes LINE, SINE, LTR and DNA. Additionally, the “FindAllMarkers” function was used to determine whether any of these TE classes were over-expressed in specific spatial regions, and its statistical significance (Supplementary File S3).

## 4. Conclusions

Spatial transcriptomics (ST) is increasing the understanding of the changes that occur in gene expression changes on a genome-wide scale across tissue sections. It is expected that as the technology advances, the uses of ST will become a common practice in cell biology research. To increase the potential of the analyses performed by ST, we developed SpatialTE, a quantitative bioinformatic tool that allows the examination and analysis of TE expression from datasets obtained by ST from tissues, such as the brain, spinal cord, kidney, etc. According to our benchmarking and validation experiment, SpatialTE can precisely pinpoint the spatial location of TE expression. Although there are a few caveats to be aware of. For example, when the TE expression was analyzed at the locus level, we found that between 12–30% of TE reads were not accurately assigned (Supplementary File S1). This was likely because (1) some TEs could be labeled as “young”, which are difficult to study using short reads (i.e., they complicate the read mapping process and posterior locus-specific read assignment), and (2) some TEs share high similarity to other subfamilies, such as the L1_Mus1 TEs, whose members are similar between them, and also similar to members of the L1Md_F2 subfamily (Figure S4).

ALS is a fatal neurodegenerative disorder in which motor neurons, present in the ventral horn of the spinal cord, die as the disease progresses in time. Unfortunately, the cellular and molecular mechanisms underlying the ALS are still unclear, although several labs have described major changes in gene expression as motor neurons get sick. We have been interested in the role that TEs play as potential regulatory elements of genes in ALS, and thus analyzed TEs expression in bulk RNAseq datasets from whole ALS spinal cords (SOD1^G93A^ mouse model). To our surprise, major changes in TEs expression, including some of their subclasses, can be observed only in ALS spinal cords, when compared to healthy spinal cords. Despite the findings, we could not determine the contribution of different cells to the overall changes in TEs expression in ALS. We believe ST can help answer the question and thus developed SpatialTE and applied it to ST datasets from ALS spinal cords at the end stage of the disease as a proof of concept. Using SpatialTE, we were able to identify TEs that were expressed in particular areas of the ALS spinal cord (ventral, medial and dorsal horn). We showed that mainly TEs of the LINE, SINE, and LTR groups contributed to the overall changes in the TEs expression, results that were consistent with our previous studies using bulk RNAseq datasets. Now, thanks to SpatialTE, we can begin to associate the TE expression with spatially identified cells in the spinal cord, ventral horn areas, dominated by motor neurons, versus medial and dorsal horn areas, enriched with specific interneurons and sensory neurons. Interestingly, our analysis shows TE expression in both ventral and dorsal horns but little change in medial regions. It is important to note that during ALS progression, and at the end stage, most motor neurons have already died (i.e., apoptosis and other mechanisms), leaving spaces filled with reactive glial cells. A similar type of gliosis has been reported to occur at the dorsal horn of ALS spinal cords. Thus, we believe that the TE changes observed show the TEs signature of reactive glial cells within the ALS afflicted spinal cord. Further temporal analysis of ST and spatial TEs are necessary to pinpoint the TE signature that belongs to motor neurons and glial cells. Therefore, SpatialTE enables analysis of the TE expression at the cellular and spatial levels, improving our knowledge about the role of TEs in gene regulation during disease.

Understanding the role of TEs in gene regulation is relevant to many other degenerative diseases that occur in the brain or in peripheral organs. As a proof of principle, we performed TE expression analysis in several brain and kidney sections. Remarkably, SpatialTE was able to detect spatial “signatures” for TEs in different regions of the brain and the kidney. The role of such differences in TEs expression is unclear, but it is possible to speculate that specific cells regulate their expression in a region-specific manner. Again, future studies will benefit from SpatialTE and begin to shed light on the mechanisms behind these differences in TEs expression, and importantly, clarify whether TEs play a role in regulating gene expression in a cell-specific manner.

## Figures and Tables

**Table 1:** Average log_2_ (fold enrichment) ^1^ TE classes across spatial regions of analyzed tissues.

Organ	Section	LINE	SINE	LTR	DNA
Brain	Coronal	0.170	−0.189	0.200	−0.245
	Sagittal Anterior 1	0.320	0.266	0.678	0.483
	Sagittal Anterior 2	0.364	0.473	0.262	0.622
	Sagital Posterior 1	0.269	0.264	0.506	0.739
	Sagital Posterior 2	0.318	0.527	0.403	0.751
Kidney	Coronal	0.729	0.311	0.519	0.253

^1^ All fold enrichments are statistically significant (adjusted *p*-value < 0.05, Supplementary File S3).

## Supplementary Materials

The following are available online at https://www.mdpi.com/article/10.3390/ijms222413623/s1.

## References

[1] Stark R., Grzelak M., Hadfield J. (2019). RNA sequencing: The teenage years. Nat. Rev. Genet..

[2] Ståhl P.L., Salmén F., Vickovic S., Lundmark A., Navarro J.F., Magnusson J., Giacomello S., Asp M., Westholm J.O., Huss M. (2016). Visualization and analysis of gene expression in tissue sections by spatial transcriptomics. Science.

[3] Fan J., Slowikowski K., Zhang F. (2020). Single-cell transcriptomics in cancer: Computational challenges and opportunities. Exp. Mol. Med..

[4] Maniatis S., Petrescu J., Phatnani H. (2021). Spatially resolved transcriptomics and its applications in cancer. Curr. Opin. Genet. Dev..

[5] Berglund E., Maaskola J., Schultz N., Friedrich S., Marklund M., Bergenstråhle J., Tarish F., Tanoglidi A., Vickovic S., Larsson L. (2018). Spatial maps of prostate cancer transcriptomes reveal an unexplored landscape of heterogeneity. Nat. Commun..

[6] Thrane K., Eriksson H., Maaskola J., Hansson J., Lundeberg J. (2018). Spatially resolved transcriptomics enables dissection of genetic heterogeneity in stage III cutaneous malignant melanoma. Cancer Res..

[7] Maniatis S., Äijö T., Vickovic S., Braine C., Kang K., Mollbrink A., Fagegaltier D., Andrusivová Ž., Saarenpää S., Saiz-Castro G. (2019). Spatiotemporal dynamics of molecular pathology in amyotrophic lateral sclerosis. Science.

[8] Lanciano S., Cristofari G. (2020). Measuring and interpreting transposable element expression. Nat. Rev. Genet..

[9] Todd C.D., Deniz Ö., Taylor D., Branco M.R. (2019). Functional evaluation of transposable elements as enhancers in mouse embryonic and trophoblast stem cells. Elife.

[10] Jin Y., Tam O.H., Paniagua E., Hammell M. (2015). TEtranscripts: A package for including transposable elements in differential expression analysis of RNA-seq datasets. Bioinformatics.

[11] Valdebenito-Maturana B., Riadi G. (2018). TEcandidates: Prediction of genomic origin of expressed transposable elements using RNA-seq data. Bioinformatics.

[12] Valdebenito-Maturana B., Torres F., Carrasco M., Tapia J.C. (2021). Differential regulation of transposable elements (TEs) during the murine submandibular gland development. Mob. DNA.

[13] Valdebenito-Maturana B., Arancibia E., Riadi G., Tapia J.C., Carrasco M. (2021). Locus-specific analysis of Transposable Elements during the progression of ALS in the SOD1G93A mouse model. PLoS ONE.

[14] Gurney M.E., Pu H., Chiu A.Y., Dal Canto M.C., Polchow C.Y., Alexander D.D., Caliendo J., Hentati A., Kwon Y.W., Deng H.X. (1994). Motor neuron degeneration in mice that express a human Cu,Zn superoxide dismutase mutation. Science.

[15] Phatnani H.P., Guarnieri P., Friedman B.A., Carrasco M.A., Muratet M., Keeffe S.O. (2013). Intricate interplay between astrocytes and motor neurons in ALS. Proc. Natl. Acad. Sci. USA.

[16] Picardi G., Spalloni A., Generosi A., Paci B., Mercuri N.B., Luce M., Longone P., Cricenti A. (2018). Tissue degeneration in ALS affected spinal cord evaluated by Raman spectroscopy. Sci. Rep..

[17] Savage A.L., Schumann G.G., Breen G., Bubb V.J., Al-Chalabi A., Quinn J.P. (2019). Retrotransposons in the development and progression of amyotrophic lateral sclerosis. J. Neurol. Neurosurg. Psychiatry.

[18] Liu E.Y., Russ J., Cali C.P., Phan J.M., Amlie-Wolf A., Lee E.B. (2019). Loss of Nuclear TDP-43 Is Associated with Decondensation of LINE Retrotransposons. Cell Rep..

[19] Li W., Lee M.-H., Henderson L., Tyagi R., Bachani M., Steiner J., Campanac E., Hoffman D.A., Von Geldern G., Johnson K. (2015). Human endogenous retrovirus-K contributes to motor neuron disease. Sci. Transl. Med..

[20] Gerdes P., Richardson S.R., Mager D.L., Faulkner G.J. (2016). Transposable elements in the mammalian embryo: Pioneers surviving through stealth and service. Genome Biol..

[21] Jachowicz J.W., Torres-Padilla M.E. (2016). LINEs in mice: Features, families, and potential roles in early development. Chromosoma.

[22] Richardson S.R., Morell S., Faulkner G.J. (2014). L1 Retrotransposons and Somatic Mosaicism in the Brain. Annu. Rev. Genet..

[23] Faulkner G.J., Billon V. (2018). L1 retrotransposition in the soma: A field jumping ahead. Mob. DNA.

[24] Misiak B., Ricceri L., Sasiadek M.M. (2019). Transposable elements and their epigenetic regulation in mental disorders: Current evidence in the field. Front. Genet..

[25] Song R., Yosypiv I.V. (2012). Development of the kidney medulla. Organogenesis.

[26] Navarro J.F., Sjöstrand J., Salmén F., Lundeberg J., Ståhl P.L. (2017). ST Pipeline: An automated pipeline for spatial mapping of unique transcripts. Bioinformatics.

[27] Navarro Gonzalez J., Zweig A.S., Speir M.L., Schmelter D., Rosenbloom K.R., Raney B.J., Powell C.C., Nassar L.R., Maulding N.D., Lee C.M. (2021). The UCSC Genome Browser database: 2021 update. Nucleic Acids Res..

[28] Quinlan A.R., Hall I.M. (2010). BEDTools: A flexible suite of utilities for comparing genomic features. Bioinformatics.

[29] Frazee A.C., Jaffe A.E., Langmead B., Leek J.T. (2015). Polyester: Simulating RNA-seq datasets with differential transcript expression. Bioinformatics.

[30] R Core Team (2021). R: A Language and Environment for Statistical Computing.

[31] Hao Y., Hao S., Andersen-Nissen E., Mauck W.M., Zheng S., Butler A., Lee M.J., Wilk A.J., Darby C., Zager M. (2021). Integrated analysis of multimodal single-cell data. Cell.

