# Supplementary material for: Spatially Resolved Expression of Transposable Elements in Disease and Somatic Tissue with SpatialTE
Source: Int J Mol Sci. 2021 Dec 20;22(24):13623. doi: 10.3390/ijms222413623 (PMC8708317; doi:10.3390/ijms222413623)
Supplement: Supplementary file 1 [file ijms-22-13623-s001.zip › Supplementary File S1.pdf]

Spatially resolved expression of Transposable Elements in disease and somatic tissue with SpatialTE

Braulio Valdebenito-Maturana, Cristina Guatimosim, Mónica Alejandra Carrasco and Juan Carlos Tapia

Supplementary File S1

Contents of this file

Supplementary methods describing simulated experiment to benchmark SpatialTE

Figures S1 to S4

Tables S1 to S2

SUPPLEMENTARY FILE S1 – SIMULATED EXPERIMENT TO TEST SPATIALTE

Spatial Transcriptomics use a paired-end sequencing layout in which the left read carries information regarding the spatial location, and the molecule identifier, and the second read carries information of the captured transcript. Taking this into account, we selected all the reads belonging to 2 spots of the sample SRR7895713: the 11x5 spot and the 10x10 spot (Figure S1). Then, we randomly selected LINEs and LTRs matching the available number of molecules at each spot. For each randomly selected TE in the respective spot, a random number of reads was generated using the Polyester RNA-Seq read simulator in single-end mode. Overall, for the spot 11x5 a total of 6420 reads were generated, while for the 10x10 spot a total of 9593 reads were generated (Supplementary Tables S1 and S2). Finally, the simulated read file was used as the right read, and the reads belonging to the spatial information of the respective spot (11x5 for LINEs, 10x10 for LTRs) was used as the left read. These files were then used as input for SpatialTE. The advantage of this experiment design is that since the spatial location and molecule info belongs to a real experiment (SRR7895713), we can use the respective H&E image to visually inspect the results of SpatialTE.

The results of this simulated experiment are shown in Figure S2 and Figures S3 and S4.

The images in Figure S2 were generated using the st_data_plotter tool, in a similar way as described in the main text (the color palette is now a sequential palette as described in a later comment). This tool allows to plot the total expression from the count file using a regular expression (*..*, Figure S2A and S2B), and belonging to a specific class in the same manner (.*LTR.* and .*LINE.*, Figure S2A' and S2B' respectively). Note that in both cases the TE expression matches the total expression, indicating that SpatialTE is able to correctly assess the spot in which TEs are expressed, and does not incorrectly assigns TEs to other spots.

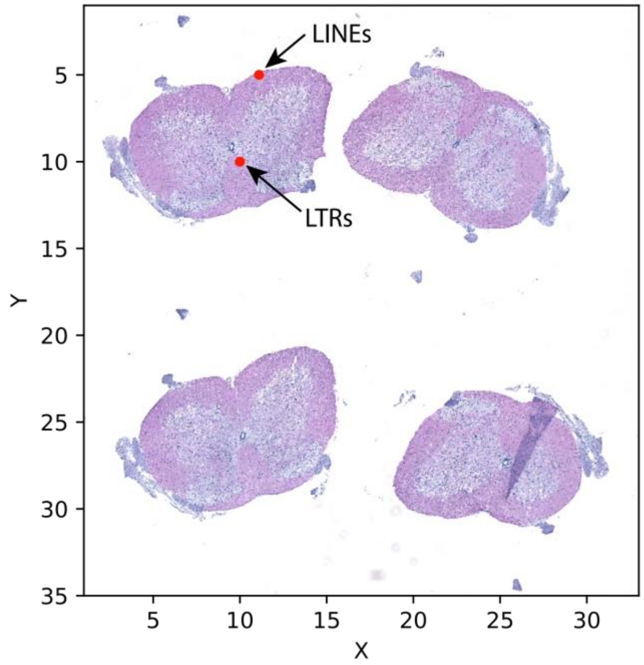

Figure S1. Selected spots for the simulation experiments. TE classes simulated at each spot are indicated.

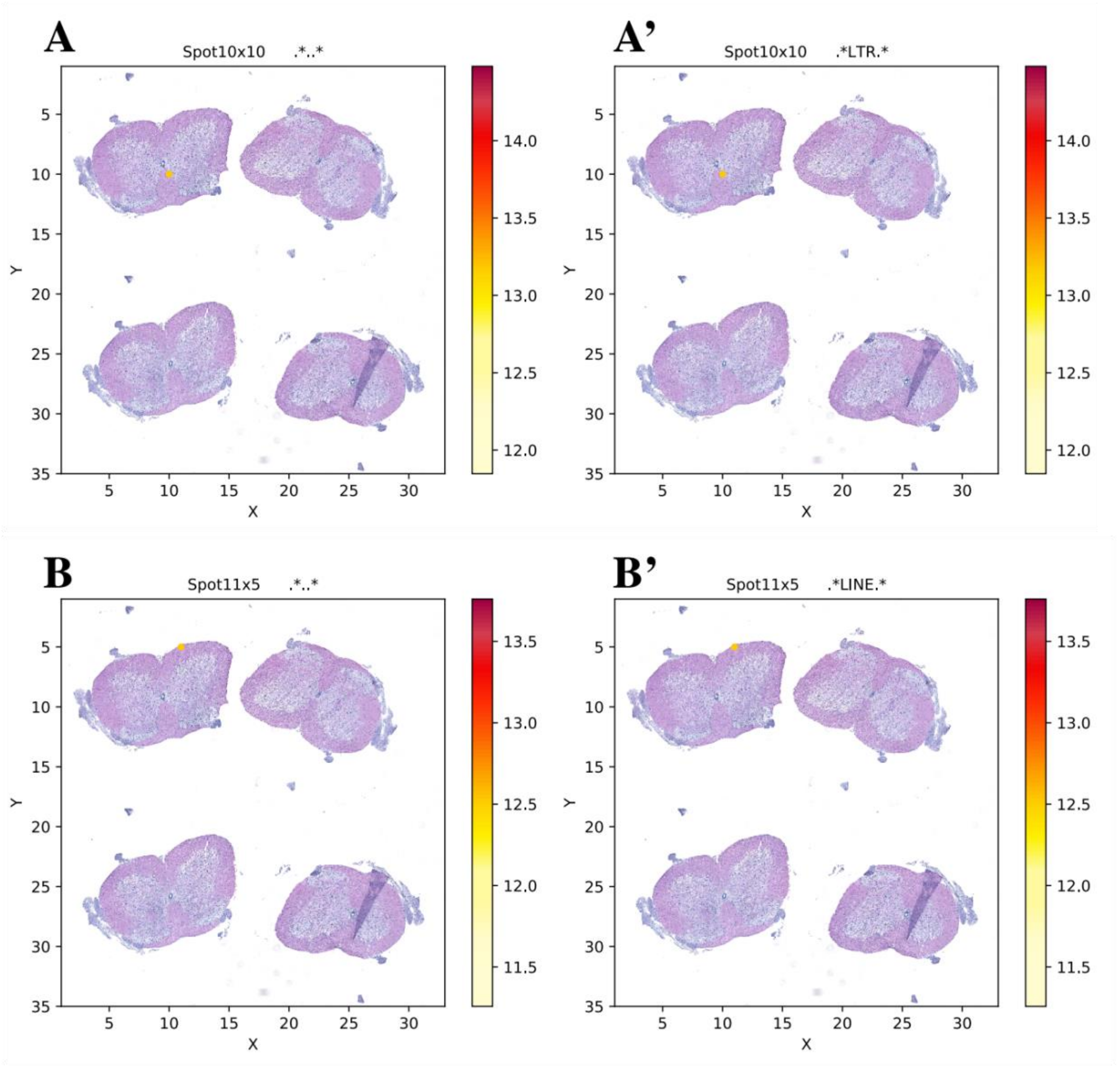

Figure S2. SpatialTE simulated experiment results shown over the H&E image. **A.** Total TE expression over the 10x10 spot. **A’**. Total LTR expression over the 10x10 spot. **B.** Total TE expression over the 11x5 spot. **B’**. Total LINE expression over the 11x5 spot.

Next, considering that with our read simulation we know the ground truth of the locus of expression of TEs, we were able to address the second question of how correct are the expression estimates obtained with SpatialTE.

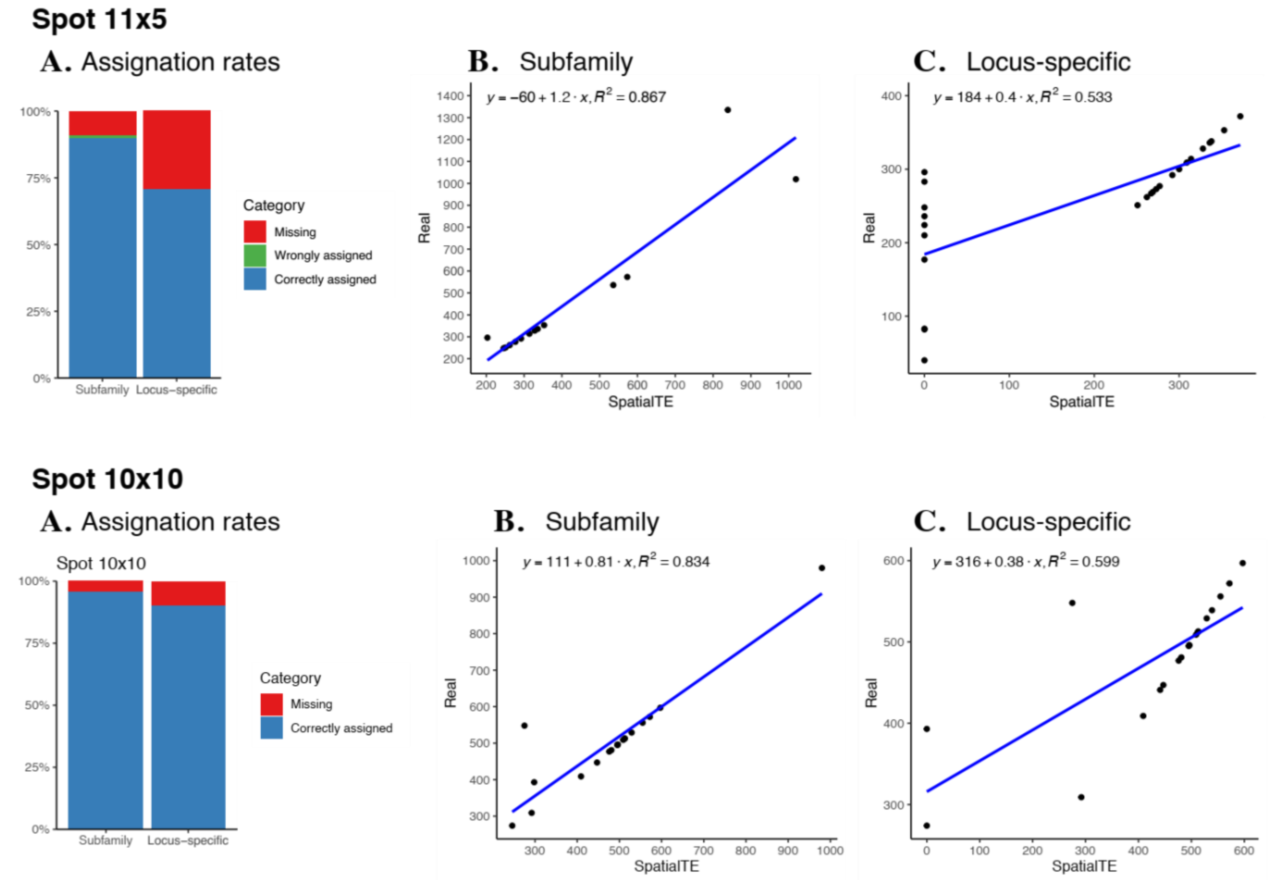

Figure S3. Upper half, results for the Spot 11x5. Second half, results for the Spot 10x10. For each read that SpatialTE was able to annotate as belonging to a TE, a comparison to the TE annotation from which the read was originated was performed. If SpatialTE was able to identify the same locus or same subfamily, it was labeled as "Correctly assignated". If SpatialTE was able to identify the reads as a TE read, but not matching the locus or subfamily it was labeled as "Wrongly assignated". If the read was not annotated with SpatialTE it was labeled as "Missing". For the Subfamilies and Locus-specific TEs "Correctly assignated", correlation plots of their expression were generated (Parts B and C).

Overall, depending on the class, between 12%-30% of TEs are lost at the locus-specific resolution. This is also reflected in the correlation plots, at x=0 where SpatialTE was not able to found an expressed TE with locus resolution. In terms of subfamily expression, the correlations seem to be better (both cases > 0.8), and the proportion of missed reads is lower. In the Spot 11x5 there seem to be over-estimation of one TE subfamily at the expense of an under-estimation of another TE subfamily. Taken collectively, these results indicate that the Subfamily results of SpatialTE seem to be the best, at the cost of losing the locus of expression of TEs.

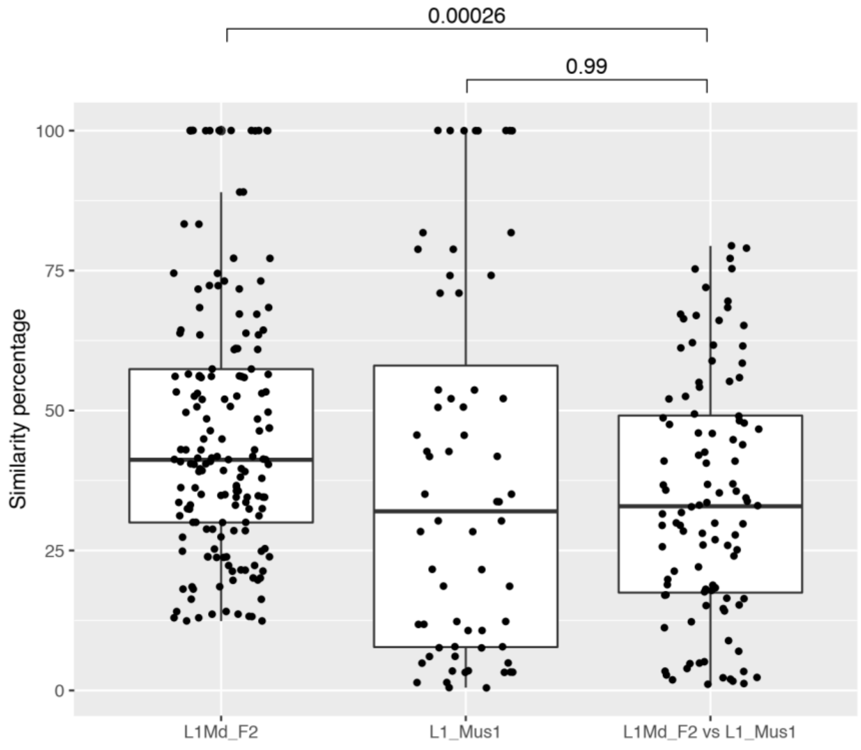

Figure S4. Box plot to show percentages of similarity between the selected subfamilies at the intra- ("L1Md_F2" and "L1_Mus1") and inter-subfamily levels ("L1Md_F2 vs L1_Mus1"). Brackets indicate comparisons from which statistical significance (numbers above brackets) was calculated.

Supplementary Table S1 - Simulated read counts for the 11x5 Spot.

TE	Reads
chr10|119991773|119992127|L1Md_F2:L1:LINE|4.5|+	82
chr10|122643555|122644004|L1ME2:L1:LINE|29.3|-	328
chr1|128814179|128814338|L1MA6:L1:LINE|23.7|-	353
chr1|142519432|142519572|L1Md_F2:L1:LINE|8.0|-	283
chr1|146813997|146814471|Lx5c:L1:LINE|28.4|-	277
chr1|159301561|159301825|Lx8b:L1:LINE|26.1|-	251
chr1|28075710|28075954|L1Md_F2:L1:LINE|4.9|+	177
chr14|93685636|93685737|L1M2:L1:LINE|26.8|-	269
chr1|58987162|58987292|L1MC:L1:LINE|26.6|-	372
chr16|42506889|42507522|L1Md_F2:L1:LINE|6.9|-	236
chr4|10518435|10518617|L1M2:L1:LINE|21.6|-	267
chr4|25310509|25311475|L1Md_F2:L1:LINE|5.0|+	224
chr5|140913677|140913886|L1ME3Cz:L1:LINE|31.2|-	262
chr6|103285931|103286589|L1Md_F2:L1:LINE|7.0|+	210
chr6|119261926|119262162|L1M5:L1:LINE|32.6|+	300
chr6|139097324|139097677|Lx5:L1:LINE|23.1|+	292
chr6|65808390|65808703|L1MC:L1:LINE|25.5|-	309
chr7|138933987|138934249|L1M5:L1:LINE|35.2|-	273
chr8|82339208|82339513|L1MC:L1:LINE|30.0|-	338
chrX|134866631|134866694|L2a:L2:LINE|32.2|+	314
chrX|137807584|137808106|L1Md_F2:L1:LINE|3.9|-	83
chrX|140961311|140961395|L1MB7:L1:LINE|23.1|-	336
chrX|70180270|70181478|L1_Mur2:L1:LINE|15.7|+	248
chrX|92293580|92293747|L1_Mus1:L1:LINE|7.7|+	296
chrY|84310454|84311022|L1Md_F2:L1:LINE|6.7|+	40
**TOTAL**	**6420**

Supplementary Table S2 - Simulated read counts for the 10x10 Spot.

**TE**	**Reads**
chr10|63424746|63424871|ORR1D2:ERVL-MaLR:LTR|20.6|-	597
chr10|79560128|79560529|MTC:ERVL-MaLR:LTR|15.8|+	477
chr1|13914612|13914829|MTEa:ERVL-MaLR:LTR|23.8|+	441
chr1|161532694|161532892|IAPEz-int:ERVK:LTR|11.0|+	274
chr11|7116698|7116785|LTR106_Mam:LTR:LTR|22.9|-	512
chr12|23849084|23850048|ORR1A0-int:ERVL-MaLR:LTR|5.6|-	393
chr13|28036655|28036746|RLTR51A_Mm:ERVK?:LTR|26.1|-	495
chr13|34102530|34102729|RLTR11C_MM:ERVK:LTR|14.8|-	447
chr14|114434392|114434507|RLTR17B_Mm:ERVK:LTR|25.7|-	309
chr15|87002191|87002483|MLT1F2:ERVL-MaLR:LTR|34.1|-	496
chr16|21006668|21007173|RLTR16:ERVK:LTR|19.2|+	556
chr17|65692530|65692641|RLTR20A4:ERVK:LTR|30.6|-	548
chr17|70734269|70734777|RMER5:ERV1:LTR|26.2|-	513
chr18|23673509|23673777|MTEa:ERVL-MaLR:LTR|24.2|+	539
chr18|83695076|83695548|RLTR28:ERVL:LTR|17.8|-	496
chr19|60181750|60182157|MT2A:ERVL:LTR|28.4|+	409
chr2|142602043|142602126|MER34:ERV1:LTR|21.7|+	509
chr2|52133617|52133714|MLT1C:ERVL-MaLR:LTR|28.6|-	572
chr5|34680657|34680784|MLT1A1:ERVL-MaLR:LTR|29.3|-	529
chrX|137262589|137262686|MLT1H2:ERVL-MaLR:LTR|29.6|-	481
**TOTAL**	**9593**
