# Supplementary material for: Spatially Resolved Expression of Transposable Elements in Disease and Somatic Tissue with SpatialTE
Source: Int J Mol Sci. 2021 Dec 20;22(24):13623. doi: 10.3390/ijms222413623 (PMC8708317; doi:10.3390/ijms222413623)
Supplement: Supplementary file 1 [file ijms-22-13623-s001.zip › Supplementary File S2.pdf]

Supplementary table - Selected Visium 10X Spatial Transcriptomics datasets

Mouse Brain Section Coronal 1	https://www.10xgenomics.com/resources/datasets/mouse-brain-section-coronal-1-standard-1-1-0
Mouse Brain Serial Section 1 Sagittal Anterior	https://www.10xgenomics.com/resources/datasets/mouse-brain-serial-section-1-sagittal-anterior-1-standard-1-1-0
Mouse Brain Serial Section 1 Sagittal Posterior	https://www.10xgenomics.com/resources/datasets/mouse-brain-serial-section-1-sagittal-posterior-1-standard-1-1-0
Mouse Brain Serial Section 2 Sagittal Anterior	https://www.10xgenomics.com/resources/datasets/mouse-brain-serial-section-2-sagittal-anterior-1-standard-1-1-0
Mouse Brain Serial Section 2 Sagittal Posterior	https://www.10xgenomics.com/resources/datasets/mouse-brain-serial-section-2-sagittal-posterior-1-standard-1-1-0
Mouse Kidney Section Coronal 1	https://www.10xgenomics.com/resources/datasets/mouse-kidney-section-coronal-1-standard-1-1-0
