# Supplementary material for: Spatially Resolved Expression of Transposable Elements in Disease and Somatic Tissue with SpatialTE
Source: Int J Mol Sci. 2021 Dec 20;22(24):13623. doi: 10.3390/ijms222413623 (PMC8708317; doi:10.3390/ijms222413623)
Supplement: Supplementary file 1 [file ijms-22-13623-s001.zip › Supplementary File S3.pdf]

# Spatially resolved expression of Transposable Elements in disease and somatic tissue with SpatialTE

**Braulio Valdebenito-Maturana, Cristina Guatimosim, Mónica Alejandra Carrasco and Juan Carlos Tapia**

**Supplementary File S3**

Contents of this file

Seurat dimensional reduction plots and statistical parameters of TEs, obtained using the “FindAllMarkers” module.

For all tables, “avg_log2FC” correspond to the Average $log_2$(Fold Changes), “p_val_adj” to the adjusted P-value, and “cluster” to the cluster in which the TE was identified as marker.

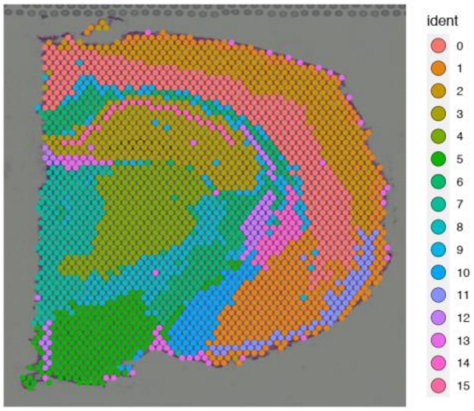


Brain coronal

| | avg_log2FC | p_val_adj | cluster |
|---|---|---|---|
| LTR | 0.20019091 | 3.73E-47 | 0 |
| SINE | -0.1886294 | 2.46E-33 | 0 |
| LINE | 0.17045444 | 1.04E-18 | 4 |
| DNA | -0.2454087 | 2.00E-05 | 5 |

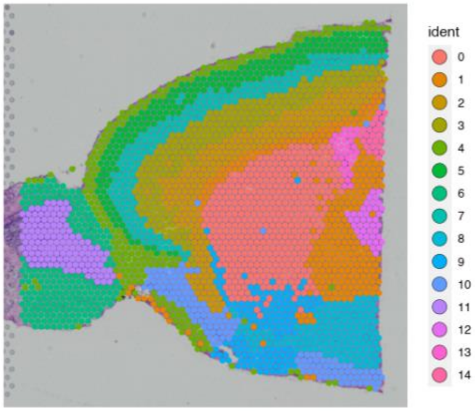


Sagittal anterior 1

| | avg_log2FC | p_val_adj | cluster |
|---|---|---|---|
| SINE | 0.26578099 | 6.31E-42 | 8 |
| LTR | 0.67821489 | 1.71E-72 | 11 |
| LINE | 0.31952677 | 3.44E-34 | 11 |
| DNA | 0.482988 | 1.09E-20 | 11 |

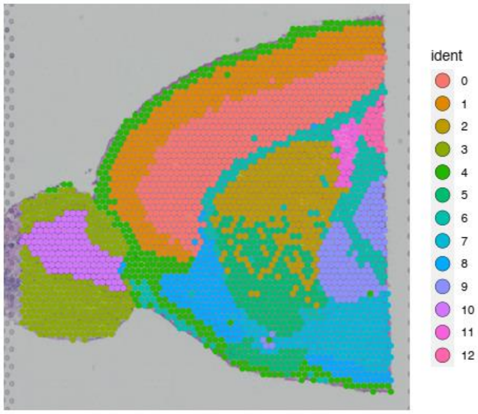


Sagittal anterior 2

| | avg_log2FC | p_val_adj | cluster |
|---|---|---|---|
| LTR | 0.26197027 | 4.30E-56 | 0 |
| LINE | 0.36430545 | 6.95E-37 | 10 |
| DNA | 0.62224674 | 7.08E-26 | 10 |
| SINE | 0.47313812 | 2.93E-12 | 12 |

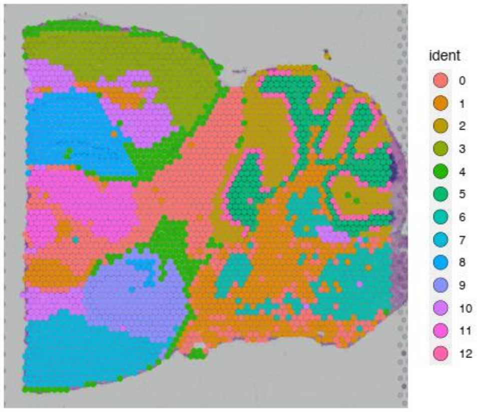


Sagittal Posterior 1

| | avg_log2FC | p_val_adj | cluster |
|---|---|---|---|
| LTR | 0.40316179 | 1.85E-45 | 2 |
| DNA | 0.75064801 | 4.85E-39 | 6 |
| LINE | 0.31805117 | 6.35E-32 | 6 |
| SINE | 0.5271769 | 6.44E-75 | 9 |

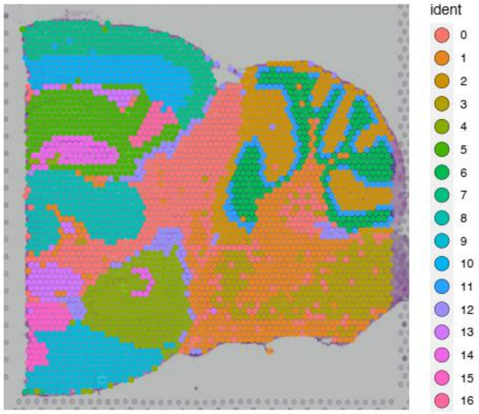


Sagittal Posterior 2

| | avg_log2FC | p_val_adj | cluster |
|---|---|---|---|
| LTR | 0.40316179 | 1.85E-45 | 2 |
| DNA | 0.75064801 | 4.85E-39 | 6 |
| LINE | 0.31805117 | 6.35E-32 | 6 |
| SINE | 0.5271769 | 6.44E-75 | 9 |

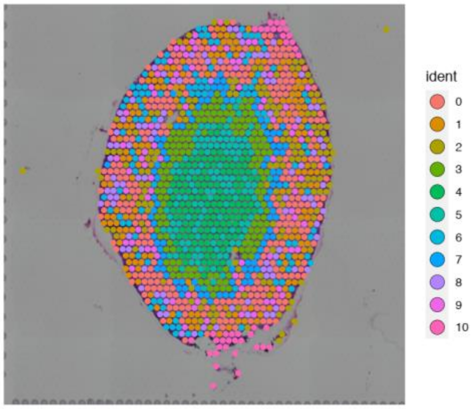


Kidney coronal

| | avg_log2FC | p_val_adj | cluster |
|---|---|---|---|
| SINE | 0.31106376 | 1.61E-16 | 1 |
| LINE | 0.72851988 | 1.19E-42 | 2 |
| LTR | 0.51897379 | 6.18E-47 | 4 |
| DNA | 0.25283316 | 0.00011998 | 4 |
